# *Campylobacter jejuni* Developed the Resistance to Bacteriophage CP39 by Phase Variable Expression of *06875* Encoding the CGPTase

**DOI:** 10.3390/v14030485

**Published:** 2022-02-26

**Authors:** Yuanyue Tang, Jie Li, Yuexuan Wang, Zhaojun Song, Hangning Ying, Linghua Kong, Xin’an Jiao, Jinlin Huang

**Affiliations:** 1 Key Laboratory of Prevention and Control of Biological Hazard Factors (Animal Origin) for Agri-Food Safety and Quality, Ministry of Agriculture of China, Yangzhou University, Wenhui East Road 48, Yangzhou 225009, China; tangyy@yzu.edu.cn (Y.T.); lijie0360@163.com (J.L.); wyx20010524@163.com (Y.W.); yhangning@163.com (H.Y.); jiao@yzu.edu.cn (X.J.); 2 Jiangsu Key Laboratory of Zoonosis/Jiangsu Co-Innovation Center for Prevention and Control of Important Animal Infectious Diseases and Zoonoses, Yangzhou University, Wenhui East Road 48, Yangzhou 225009, China; 3 Joint International Research Laboratory of Agriculture and Agri-Product Safety, Yangzhou University, Wenhui East Road 48, Yangzhou 225009, China; 4 The Fourth Affiliated Hospital, Zhejiang University School of Medicine, No.1 Shang Cheng Avenue, Yiwu 322000, China; 8619003@zju.edu.cn; 5 Department of Quality and Safety Control, Heyi Food Co., Ltd., Zaozhuang 277500, China; klh6668@126.com

**Keywords:** bacteriophage resistance, *Campylobacter jejuni*, CPS, phage resistance

## Abstract

Bacteriophage (phage) is regarded as an antimicrobial alternative for *Campylobacter* in food production. However, the development of phage resistance to the host is a main concern for the phage application. This study characterized the phage CP39 and investigated the phage resistance of CP39 in *Campylobacter jejuni* NCTC12662. We determined that phage CP39 belonged to the *Myoviridae* family by the WGS and phylogenetic analysis. Phage CP39 was confirmed as a capsular polysaccharide (CPS)-dependent phage by primary *C. jejuni* phage typing. It was further confirmed that the phage could not be adsorbed by the acapsular mutant Δ*kpsM* but showed the same lytic ability in both the wild-type strain NCTC 12662 and the Δ*motA* mutant lacking motile flagella filaments. We further determined that the *06875* gene encoding CDP-glycerol:poly (glycerophosphate) glycerophosphotransferase (CGPTase) in the CPS loci was related to phage CP39 adsorption by SNP analysis and observed a rapid development of phage resistance in NCTC 12662 during the phage infection. Furthermore, we observed a high mutation frequency of *06875* (32%), which randomly occurred in nine different sites in the gene according to colony PCR sequencing. The mutation of the *06875* gene could cause the phase variable expression of non-functional protein and allow the bacteria against the phage infection by modifying the CPS. Our study confirmed the *06875* gene responsible for the CPS-phage adsorption for the first time and demonstrated the phase variable expression as a main mechanism for the bacteria to defend phage CP39. Our study provided knowledge for the evolutionary adaption of bacteria against the bacteriophage, which could add more information to understand the phage resistance mechanism before applying in the industry.

## 1. Introduction

Bacteriophages (or phages) play an essential part in shaping the bacterial ecosystem by the lytic process, while bacteria usually react constantly to promote a defense action during phage infection [1]. For all bacteriophages, the approach to infect bacteria starts by recognizing the specific receptor and attaching the phage particle to the bacterial surface via a receptor-binding protein (RBP) on the tail. By contracting the tail sheath, the phage contracts the tail to penetrate the cell membrane by lyases, injecting phage DNA into the cell, and using the host materials for proliferation. However, the constant phage exposure promotes bacteria to develop mechanisms specially against phage infection. The phage resistance mechanisms can either be the external prevention of phage to inject DNA by blocking or modifying phage binding receptors on the cell membrane or the internal degradation of the foreign phage genomic DNA, including the restriction–modification systems, abortive infection systems, and the CRISPR-cas interference [2]. For bacteria, the initial defense strategy is to modify the phage receptor and prevent phage absorption, which is also the most common bacterial resistance strategy during phage–host interaction [2]. Phage receptors in Gram-negative bacteria include the outer membrane proteins, lipopolysaccharides (LPS), capsular polysaccharides (CPS), pili, and flagella, which unique surface structures could ensure phage binding to the correct host [3].

*Campylobacter jejuni* is the commensal bacteria in poultry, and it is also one of the most common foodborne pathogens that cause bacterial gastroenteritis in humans worldwide. *C. jejuni* are transmitted to humans by eating uncooked meat products or directly contacting livestock during food production. Humans can be infected by eating poultry contaminated with *C. jejuni*, and threaten public health [4,5]. Bacteriophage is one of the alternatives against *C. jejuni* contamination in food production [6]. *C. jejuni* phages are known to co-exist with their host and could commonly be isolated from chicken feces and poultry-related production environments [7].

Most *C. jejuni* phages belong to the *Myoviridae* family, which are divided to group I (320–425 kbp), group II (175–183 kbp), and group III (131–135 kbp) according to genome size. *C. jejuni* phage receptors include flagella and capsular polysaccharides (CPS), while the loss of flagella motility or CPS can lead to the development of phage resistance [8,9]. A previous study has demonstrated that the flagella-dependent phage F341 attached to the flagella and required the motility for phage binding to *C. jejuni*, while the absence of flagella motility would prevent the phage attachment [10]. *C. jejuni* could defend CPS-dependent phage by phase variable expression of the phage receptor in the CPS loci. The O-methyl phosporamidate (MeO*P*N) transferase encoded by *cj1421* is involved in CPS biosynthesis, which is also responsible for the F366 phage binding in *C. jejuni* NCTC 11168. The frameshifting expression of *cj1421* due to the addition of guanine in poly G tract could allow the host to resist against the phage F366 [11]. In addition, it has been shown that the infection of CPS-dependent phages was more than the presence of MeO*P*N, indicating the diverse mechanisms of the initial phage interaction with *C. jejuni* [12].

*C. jejuni* NCTC12662 is widely used for phage isolation due to its sensitivity to many phages. The MeO*P*N encoded by the *06875* gene in NCTC 12662 served as a phage receptor for phage F207. The shift-off of this gene by poly G phase variation could help the bacteria rapidly develop phage resistance [12]. This study isolated a phage CP39 from sewage in the retail market of Yangzhou, China, using *C. jejuni* NCTC12662 as the host. After the phenotypic and genotypic characterization of phage CP39, we investigated the development of resistance mechanisms in NCTC 12662 during the phage infection. Our study demonstrated the diverse resistant mechanisms in *C. jejuni* during the phage–host interaction.

## 2. Materials and Methods

### 2.1. Bacterial Strains, Bacteriophage, Plasmids and Culture Conditions

All bacterial strains and plasmids used in this study are listed in Table S1. Briefly, *C. jejuni* were grown on Mueller-Hinton (MH) agar (BD Biosciences, Sparks, NV, USA) supplemented with 5% sheep blood or *Campylobacter* blood-free selective agar containing charcoal cefoperazone deoxycholate (CCDA) (Oxoid, Basingstoke, UK), and incubated at 42 °C under microaerobic conditions (85% N_2_, 10% CO_2_, 5% O_2_). *E. coli* DH5α strains were inoculated on Luria-Bertani (LB) agar (Sangon Biotech, Shanghai, China) at 37 °C. The pMD-20T was used as a suicide plasmid in the construction of mutant strains. The pRK2013 is a helper plasmid for triparental mating conjugation, while pUOA18 and pRY107 are *C. jejuni* shuttle vectors. *C. jejuni* phage CP39 was originally isolated from sewage in a retail market from Yangzhou, China and is propagated on *C. jejuni* NCTC12662. Phages were stored in SM buffer (0.05 M Tris–HCl, pH 7.5 with 5.8 g NaCl, 2.0 g MgSO_4_·7H_2_O, and 5 mL gelatin, 2% *w*/*v* solution) at 4 °C.

The phage propagation was performed as previously described with minor modification [12]. Briefly, the host strain *C. jejuni* NCTC 12662 was grown overnight on CCDA plate and harvested by BHI broth (BD Biosciences, Sparks, NV, USA) supplemented with 10 mM MgSO_4_ and one mM CaCl_2_ (cBHI) before adjusting the suspension to OD_600_ of 0.35. The suspension was incubated at 37 °C for 4 h under microaerobic conditions and mixed with phage CP39 at a multiplicity of infection (MOI) of 0.01. The mixture was incubated at 37 °C for 15 min for phage absorption, and with 5 mL NZCYM (Sangon Biotech, Shanghai, China) and 0.6% agar before pouring onto the NZCYM 1.2% agar plate and incubating at 42 °C for 4 h under microaerobic condition. The overlay agar was collected, vortexed with 5 mL SM buffer, and centrifuged at 8500 rpm, 4 °C for 10 min. The supernatant was filtered through a sterile 0.22-µm membrane filter.

### 2.2. Bacteriophage Titration

Bacteriophage titration was performed as described previously [13]. *C. jejuni* was inoculated on MH agar with 5% sheep blood and incubated at 42 °C overnight under microaerobic conditions before suspending in cBHI broth. The bacterial culture was adjusted to OD_600_ of 0.35 and incubated at 42 °C for 4 h under microaerobic conditions. The bacteriophage titer was evaluated by the double-layer agar plate assay, whereby 100 µL recipient strain with OD_600_ of 0.5 was mixed with 100 µL serially diluted phage stock in 5 mL soft agar and poured on a NZCYM agar plate for the count of phage plaques. For the spot assay, phage stock was serially diluted in SM buffer before 10 μL phage stock was spotted on the soft agar plate with 100 µL recipient strain NCTC 12662 with OD_600_ of 0.5 and incubated at 42 °C for 24 h under microaerobic conditions.

### 2.3. Transmission Electron Microscopy (TEM) of Phage CP39

TEM analysis was performed as previously described with modifications [14]. One hundred µL phage stock was dropped onto a mica sheet (~3 × 3 mm size) and absorbed for 10 min. The sample was stained with 2% (*w*/*v*) uranyl acetate and virtualized with the transmission electron microscope (Tecnai 12; Philips; The Netherlands).

### 2.4. Phage Genome Sequencing and Phylogenetic Analysis

Phage DNA was extracted with the phage titer of 10^8^–10^9^ PFU/mL using the ABigen λ Phage DNA Purification kit (ABigen Corporation, Beijing, China). The phage genome was sequenced by Hiseq sequencer (Illumina Inc., San Diego, CA, USA) and assembled by Newbler 2.9 (Roche Diagnostics, Branford, CT, USA). Phage CP39 genome was submitted to European Nucleotide Archive (ENA) database with the accession number MH107028. The phylogenetic analysis of phage CP39 was based on the major capsid protein by CLC genenomic workbench. All reference strains for the phylogenetic analysis are listed in Table S2.

### 2.5. Construction of C. jejuni Mutant and Complemented Strains

Δ*motA*, Δ*kpsM*, Δ*flaAB*, Δ*06810,* and Δ*06875* mutants were constructed in *C. jejuni* NCTC12662 by replacing target genes with *aphA* encoding the kanamycin-resistant cassette or *cat* encoding chloramphenicol resistance as previously described (Table S1) [14]. In brief, the up and down flanking regions of *motA*, *kpsM*, *flaAB*, *06810*, *06875,* and kanamycin resistant gene *aph* were amplified by PCR using the primers list in Table S3. PCR fragments and pMD-20T were ligated together by a One Step Cloning Kit (Vazyme, Nanjing, China). The recombinant plasmid was electroporated into *C. jejuni* competent cell, inoculated on CCDA agar with 50 µg/mL kanamycin, and incubated at 42 °C for 24 h under microaerobic conditions. All mutants were confirmed by PCR sequencing, and the detailed information is listed in Table S1.

The *06875* complement strain was constructed by amplifying the *06875* gene by primers *06875*-pUOA18-F/R and ligating downstream of the promoter Pmetk in pUOA18. The pUOA18-pmetK-*06875* was transferred to the recipient strain NCTC12662 Δ*06875* by triparental mating with the help of plasmid pRK2013. The mixture was inoculated on MH agar with chloramphenicol (20 µg/mL), polymyxin B (6.7 µg/mL), rifampicin (10 µg/mL), and trimethoprim (5 µg/mL), and incubated at 42 °C for 24 h under microaerobic conditions [15]. The complement strain was confirmed by PCR sequencing and named as Δ*06875*::P*_06875_* (Table S1).

The point mutation of *06875* gene was constructed by amplifying the gene with primers 559A-up-F/R and 559A-down-F/R containing mutant basepairs and ligating downstream of the promoter Pmetk in pUOA18. The pUOA18-pmetK-*06875*-559A was mobilized into *06875* mutant strain by triparental mating with the help of the pRK2013, and inoculated on MH agar with chloramphenicol (20 µg/mL), polymyxin B (6.7 µg/mL), rifampicin (10 µg/mL), and trimethoprim (5 µg/mL). The successful mutant was confirmed by sequencing and the mutant was named as *06875*-492delT.

### 2.6. Bacteriophage Sensitivity Assay

*C. jejuni* NCTC12662, Δ*motA*, Δ*kpsM*, Δ*flaAB*, ∆*06810*, Δ*06875*, Δ*06875*::P*_06875_*, and *06875*-492delT sensitivity to phage CP39 was evaluated by the double-layer agar plate assay as described previously [7]. Briefly, wild-type *C. jejuni* NCTC12662 or its mutant strains with the OD_600_ of 0.5 was mixed with soft agar and poured on NZCYM agar plate. After solidification, 10 μL phage stock was spotted on the bacterial lawns and incubated at 42 °C for 24 h under microaerobic conditions. The sensitivity of phage CP39 was evaluated by the plaque formation (PFU/mL) on the soft agar.

### 2.7. Bacterial Growth Curve

Overnight cultures of *C. jejuni* NCTC12662, Δ*motA* and Δ*kpsM* mutant strain were suspended and diluted in MH broth with OD_600_ value of 0.07. Ten mL of the cultural suspension was incubated at 42 °C and 100 rpm under microaerobic conditions. The OD_600_ value of the *C. jejuni* broth culture was measured every 4 h until 24 h. Each experiment was performed in triplicate.

### 2.8. Bacteriophage Adsorption Assay

A bacteriophage adsorption assay was performed as previously described [16]. Briefly, phage CP39 was mixed with *C. jejuni* NCTC12662, Δ*motA,* and Δ*kpsM* in cBHI broth with an MOI of 0.01, respectively. After incubation at 42 °C with 100 rpm for 90 min, the mixture was centrifuged at 8500 rpm for 10 min, then filtered through an 0.22 μm pore diameter membrane filter. The adsorption level was determined by measuring bacteriophage titration. Each experiment was performed in triplicate.

### 2.9. Isolation of Phage-Resistant Variants

A hundred μL of CP39 phage suspension with 10^6^ PFU/mL were inoculated in 5 mL NZCYM with 0.6% of agar and poured onto the NZCYM agar plate to create a phage lawn. *C. jejuni* NCTC12662 in cBHI broth was adjusted to OD_600_ of 0.35 and incubated at 42 °C for 4 h under microaerobic conditions. Subsequently, *C. jejuni* was serially diluted from 10^−1^ to 10^−4^ in SM buffer. Ten μL suspension was spotted on the phage lawn and incubated under microaerobic conditions until colonies were produced on the plaques. Single colonies were tested for resistance against CP39 by plaque assay. The suspicious resistant colonies were re-streaked five times on MH agar plates, and the resistance of colonies against CP39 was tested after each time by plaque assay. After being re-streaked five times on MH agar, the single colony conferring phage resistance was regarded as *C. jejuni* strain resistant to phage CP39. Four isolates with resistance to CP39 were randomly selected for whole-genome sequencing and named as 12662_CP39R1, 12662_CP39R2, 12662_CP39R3, and 12662_CP39R4.

### 2.10. Bacterial Genome Sequencing and Analysis

Genomic DNA was extracted from 12662_CP39R1, 12662_CP39R2, 12662_CP39R3, and 12662_CP39R4 using TIANamp Bacteria DNA Kit (Tiangen, Beijing, China) and sequenced by Illumina Hiseq 2500. Reads were assembled to contigs by SPAdes 3.12. SNPs were analyzed by mauve and mapped to the NCTC12662 reference genome with accession number NZ_CP019965 in the NCBI database. Sequences of four phage resistant isolates were uploaded to the NCBI database with the accession number PRJNA798540. Genes with SNPs from the four phage resistant isolates are listed in Table S4.

### 2.11. Phage-Bacteria Co-Cultivation and the One-Step Phage Growth Curve

Phage-resistant populations were generated via co-culture of phage and *C. jejuni*. Overnight cultures of *C. jejuni* were harvested and diluted in cBHI broth with an OD_600_ value of 0.07. CP39 was added at MOI of 0.01, and co-culture was incubated at 42 °C 100 rpm. The OD_600_ value was measured at each hour until 6 h and each 6 h until 36 h. The one-step phage growth was also performed during the phage–host co-cultivation. At each time point, 500 μL samples were centrifuged at 8500 rpm, and supernatants were filtered through a 0.22-μm diameter pore membrane filter, and the concentration of phage was evaluated by PFU/mL.

### 2.12. The Mutation of 06875 in NCTC12662 during Phage Infection

After 36 h co-cultivation of phage CP39 and *C. jejuni* NCTC12662, the mixtures were serially diluted to 10^−4^ in PBS, and 100 μL of each dilution was spread on the CCDA plate and incubated at 42 °C for 24 h under microaerobic conditions. One hundred colonies were selected for the PCR amplification of the *06875* with primers *06875*-outer-F and *06875*-outer-R (Table S3). The PCR products were sequenced by Sangon Biotech and analyzed for mutations of the *06875* gene by NCBI BLAST. The mutation rate of *06875* was calculated as the number of *06875* mutants divided by the total sequenced colonies.

## 3. Results

### 3.1. Identification and Characterization of Phage CP39

**Figure 1:**
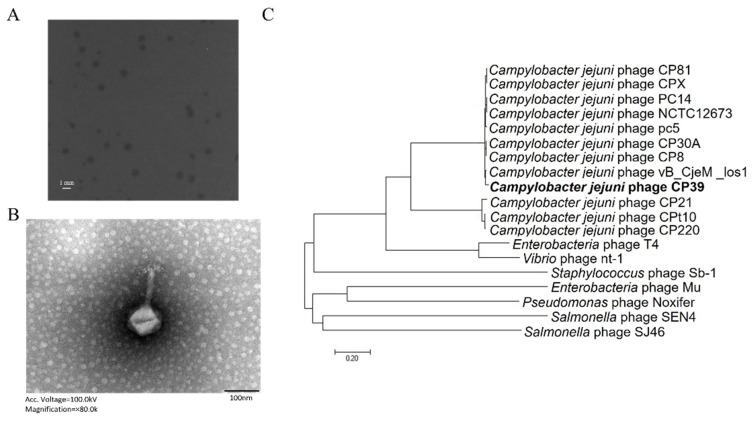
Identification and characterization of *Campylobacter jejuni* phage CP39. (**A**) The plaque morphology of *C. jejuni* phage CP39. (**B**) transmission electron microscopy (TEM) image of phage CP39 indicating the phage belongs to *Myoviridae* family. (**C**) The phylogenetic relationship of phage CP39. Additional information of genomes was listed in Table S2.

Phage CP39 was isolated from the sewage of a retail market in Yangzhou, China. The phage formed circulated and transparent plaques with 1–2 mm (Figure 1A). TEM showed that phage CP39 belonged to the *Myoviridae* family with a head size of approximately 80 nm and connected to a terminal bleb at the distal ends of their tails (Figure 1B). Genome sequencing showed that the whole genome size of phage CP39 was 130,715 bp containing 168 ORF and three tRNAs. The phylogenetic analysis revealed that phage CP39 was clustered into *Fletchervirus* phages as reference strain NCTC12673 and was closely related to the phage vB_CjeM_los1, indicating phage CP39 as a CPS-dependent phage [17].

### 3.2. The Primary Determination of CP39 Phage as a CPS-Dependent Phage

**Figure 2:**
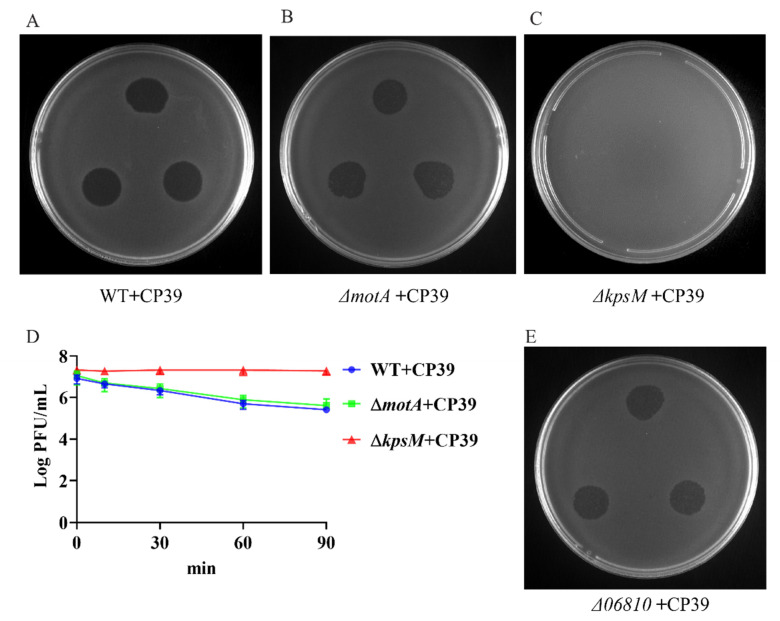
Identification of phage CP39 receptor type. (**A**) Sensitivity of NCTC12662 strain to CP39. (**B**) Sensitivity of *motA* mutant strain to CP39. (**C**) Sensitivity of *kpsM* mutant strain to CP39. (**D**) The adsorption assay of CP39 to NCTC12662, *motA* and *kpsM* mutant strain. *motA* and *kpsM* mutant strains were infected with CP39 for 100 min at an MOI of 0.01. The same culture NCTC12662 served as the control. (**E**) Sensitivity of Δ*06810* mutant to CP39.

According to the previous study, we primarily investigated the phage type and its receptor based on the genomic analysis [7]. We constructed NCTC12662 Δ*motA* mutant without motile flagella filaments and an acapsular *kpsM* mutant NCTC12662 Δ*kpsM* for phage typing. Both Δ*motA* and Δ*kpsM* mutants showed not significant growth differences from the wild type (WT) NCTC12662 (Figure S1). The receptor type of CP39 was determined by evaluating plaques formation of CP39 on the soft agar plate containing wild type, Δ*motA* or Δ*kpsM* mutant. Our results demonstrated that CP39 could form a clear plaque on wild type (Figure 2A) and Δ*motA* mutant (Figure 2B), while no plaque was observed on Δ*kpsM* (Figure 2C), indicating that the absence of CPS in the recipient strain could influence the phage adsorption. We also investigated the adsorption of phage in liquid broth and observed a 2 LogPFU/mL decrease of free phage in wild type and Δ*motA* mutant, while the free phage in Δ*kpsM* mutant remained stable (Figure 2D). In addition, we also constructed the mutant Δ*flaAB* lacking the flagellar filaments and confirmed that the phage infection did not involve the flagellar (Figure S2A). The above result demonstrated that phage CP39 was a CPS-dependent phage.

Previous studies have reported the MeO*P*N -transferase is responsible for the infection of CPS-dependent phage in *C. jejuni* [12,18,19]. Therefore, we constructed mutant of the *06810* gene encoding the MeO*P*N-transferase in NCTC12662 and conducted the phage sensitivity assay to investigate if MeO*P*N-transferase was responsible for the infection of phage CP39. As a result, we observed a clear plaque on the soft agar plate with Δ*06810* mutate as the recipient strain (Figure 2E), indicating that the receptor of phage CP39 was not dependent on the *06810* gene, but might relate to other genes located in the CPS loci.

### 3.3. The 06875 Gene Located in CPS Cluster of NCTC12662 Is Responsible for the Phage CP39 Infection

We obtained the phage-resistant isolate by spotting serially diluted NCTC12662 strain on soft agar plate containing 10^6^ PFU phage CP39, and isolated the colonies occurred on the phage lawn. Four phage-resistance isolates were randomly selected and the whole genome sequenced, including 12662_CP39R1, 12662_CP39R2, 12662_CP39R3 12662_CP39R4. After Mauve analysis, we identified several SNPs, including the B2K12_RS06420 (12662_CP39R1) encoding a motility accessory factor, B2K12_RS06335 (12662_CP39R1) and B2K12_RS06385 (12662_CP39R4) encoding flagellin modification protein PseD, B2K12_RS03815 (12662_CP39R2) encoding a hypothetical protein, B2K12_RS*06875* (12662_CP39R3) encoding CDP-glycerol-glycerophosphate glycerophosphotransferase (CGPTase) in the CPS loci, and B2K12_RS06565 (12662_CP39R4) encoding a nucleotidyltransferase (Table S4). The SNP that occurred in *06875* was one deletion of thymine (T) in a poly T tract with eight Ts in NCTC12662 (1,355,914~1,355,921 bp).

**Figure 3:**
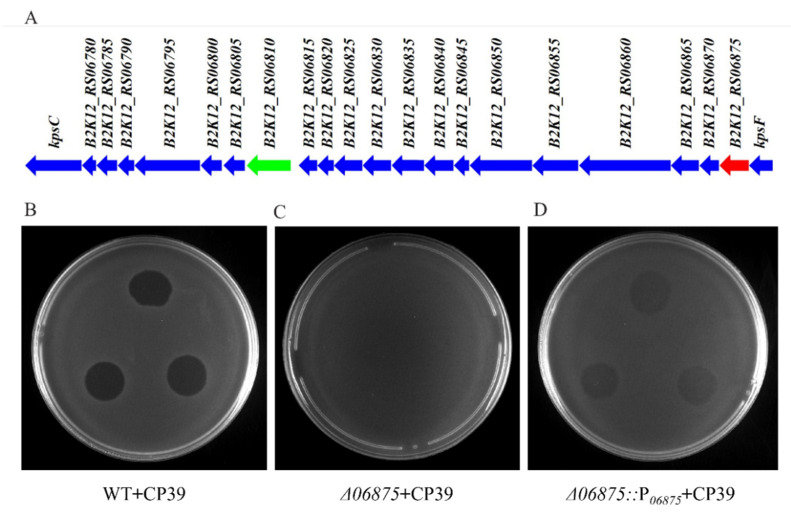
The *06875* gene in CPS loci is responsible for the CP39 resistance in *C. jejuni* NCTC12662. (**A**) Schematic diagram of CPS loci from *C. jejuni* NCTC12662. B2K12_RS*06810* (green arrow) encodes the MeO*P*N-GalfNAc transferase, which the modification of MeO*P*N is known to be responsible for *C. jejuni* phage resistance. B2K12_RS*06875* (red arrow) is annotated as CDP-glycerol-glycerophosphate glycerophosphotransferase, which related to the phage CP39 resistance in this study. The phage sensitivity assay confirmed the *06875* gene was responsible for infection of phage CP39, which wild type strain NCTC 12662 (WT) (**B**) Δ*06875* mutant (**C**) and its complementary strain Δ*06875*::P*_06875_* (**D**) was infected by CP39 on soft agar plate.

The phage resistance caused by the mutation of *06875* was further confirmed by the deletion mutant Δ*06875* and its complement, Δ*06875*::P*_06875_*. Both WT and complement strain Δ*06875*::P*_06875_* were sensitive to phage CP39 (Figure 3B,D), while the Δ*06875* mutant showed resistance to the phage infection (Figure 3C). In addition, the adsorption assay also showed that the *06875* complementary strain could partially recover the phage infection compared to the Δ*06875* mutant (Figure 3D). The above results indicated that the *06875* gene was responsible for the infection of phage CP39, while the truncated expression of *06875* could cause bacterial resistance to the phage.

### 3.4. High Mutation Frequency of 06875 Can Benefit the Bacteria to Resist Phage CP39

**Figure 4:**
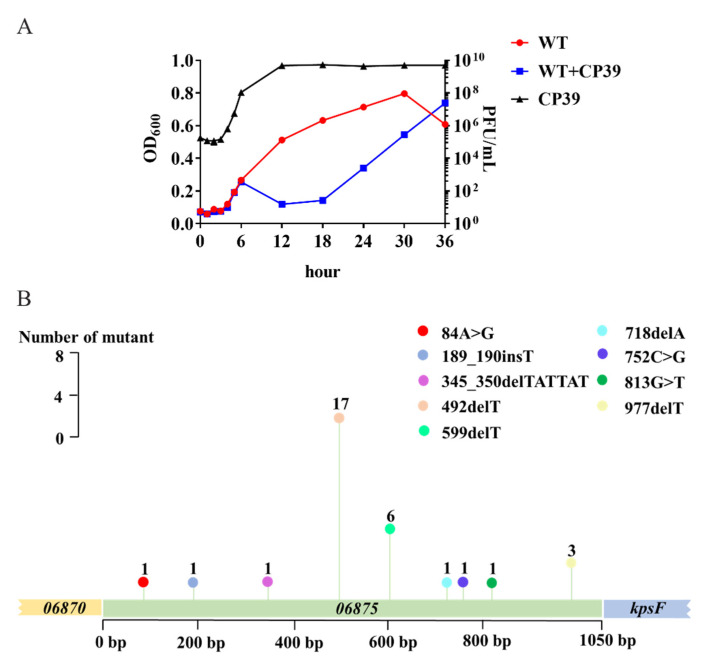
The occurrence of phage resistance in *C. jejuni* NCTC12662 and the mutation frequency of the *06875* gene during CP39 infection. (**A**) Generation of phage lysis curves and phage-resistant bacterial outgrowth. NCTC12662 was infected with CP39 for 36 h at an MOI of 0.01. NCTC12662 without phage was served as the wild type (WT) control. The one-step growth curve of phage CP39 was evaluated by the PFU/mL at each time point. (**B**) The mutation frequency of the *06875* gene. The mutation frequency was calculated as the number of *06875* mutant from 100 phage-resistant colonies by PCR sequencing. A “>” represented the original base pair was replaced by another base pair; A “_” represented an insertion (ins) or deletion (del) was occurred between base pairs.

The development of CP39 phage resistance in *C. jejuni* NCTC12662 was evaluated by the co-culture assay in cBHI broth. The growth of *C. jejuni* was suppressed by the infection of phage CP39 until 18 h. After 18 h, *C. jejuni* started to proliferate, indicating that the phage resistance subpopulation already occurred during co-cultivation. After 36 h, the antimicrobial effect of CP 39 was eliminated, while the phage-treated group entered the exponential growth phase with the OD_600_ close to control group (Figure 4A). At each time point, the concentration of CP39 was evaluated by the plaque assay. The results demonstrated that after 12 h the phage growth remained in stationary phase (Figure 4A).

After 36 h co-cultivation of phage CP39 and *C. jejuni* NCTC12662, the mixture was serially diluted on the CCDA plate, and 100 colonies were randomly selected for PCR sequencing of *06875*. Nine mutation sites were detected in the *06875* gene, with a mutation frequency of 32 (Figure 4B). The most frequent mutation (*n* = 17) was at the 492/1050 bp, in which the deletion of T occurred. Besides the most frequent mutation site, the deletion or insertion of T also occurred at 599/1050 bp (*n* = 6), 977/1050 bp (*n* = 3), and 189/1050 bp (*n* = 1) (Figure 4B). Other mutations included the deletion of a short sequence between 345 and 350/1050 bp and the deletion of one adenine (A) at 718/1050 bp, the replacement of A to G at 84/1050 bp (*n* = 1), cytosine (C) to G at 752/1050 bp (*n* = 1), and G to T at 813/1050 bp (*n* = 1) (Figure 4B). To confirm the occurrence of phage resistance in *C. jejuni* NCTC 12662, we further constructed a T-deletion mutant at 492/1050 bp in *06875*, which was the highest mutation frequency according to the PCR sequence result. No phage plaques were observed on bacterial lawns of *06875*-492delT point mutant after phage CP39 infection (Figure S2B). The above result demonstrated that the high mutation frequency of *06875* during phage CP39 infection plays an essential role in the evolution of a phage-resistant subpopulation.

## 4. Discussion

The application of bacteriophages to reduce the contamination of foodborne pathogens has been a hotspot in recent years. However, the frequent occurrence of phage resistance has prevented the application of phage as antibacterial agents for feed and food production [20,21,22]. We characterized phage CP39 and investigated the development of phage resistance in *C. jejuni* during CP39 infection. The phage morphology and the WGS analysis demonstrated that phage CP39 was closely related to the *C. jejuni* phage Los 1 (Figure 1B,C). Both phage CP39 and Los1 belonged to the family Myoviridae with a similar genome size of 130,715 bp and 134,073 bp, respectively, indicating that phage CP39 belonged to Group III phage of the Cp8unalikevirus [17,23,24]. The phylogenetic analysis of CP39 from other studies has also proven our results in that CP39 was cluttered to the group III *Campylobacter* phage [25,26]. The group III phage was known to exhibit a strong lytic activity, and its successful application could effectively decrease the contamination load *C. jejuni* in chickens [27].

The most common strategy for bacteria to defend phages is the modification of the phage receptors. We primarily determined the receptor of CP39 according to a previous study that grouped *Campylobacter* phages to the CPS-dependent or motile flagella-dependent phages [7]. Phage CP39 was primarily confirmed as CPS-dependent phage by the adsorption of phage in the wild type NCTC12662 and Δ*motA* mutant, but not the Δ*kpsM* mutant [28]. It is known that the phage–host interaction occurs beyond the cell surface. A recent study demonstrated that the group III phage NCTC 12673 required flagellar motility for the infection but did not need the flagella for the phage adsorption to the cell surface. Based on the previous studies, we constructed a Δ*flaAB* mutant lacking flagellar filaments, which was sensitive to the phage infection, indicating that the motility or flagellum was not necessary for phage CP39 to infect bacteria [29].

The phase variation of the phage receptors helps the bacteria to resist against phages in a reversible manner, which could preserve the biological function of the population and develop the phage resistant sub-population for survival [12,30]. MeO*P*N encoding by the *06810* gene has been known as a receptor of NCTC12662 for several CPS-dependent phages [12]. The switch in the length of poly G tract in the *06810* gene results in the expression of a non-functional protein of MeO*P*N and allowed the *C. jejuni* to resist against the phage [12]. A similar phenomenon was also observed in NCTC 11168, where poly G regulated phase variation occurred in both the *cj1421* gene encoding MeO*P*N transferase and the *cj1426* gene encoding the 6-*O*-Me transferase to defend the CPS-dependent phage infection [19]. We identified the genes relating to the adsorption of phage CP39 by SNP analysis, and one isolate, 12662_CP39R3, contained a deletion of T in the *06875* gene encoding CGPTase in CPS loci. The deletion of T was in a poly T tract (8× Ts) and caused the truncated expression of *06875* and influenced the adsorption of phage CP39. CGPTase is known to be involved in the synthesis of the wall teichoic acid (WTA) by transferring the glycerol-phosphate units from CDP-gro to an acceptor and forming polyglycerol-phosphate chains attached to the linkage unit lipid [31,32]. The phase variable expression of *06875* may cause the lack of CDP-glycerol units during the WTA synthesis, leading to the resistance to phage CP39. The phage resistance of the Δ*06875* mutant and the recovery of sensitivity in its complement strain confirmed that the phage CP39 required the CGPTase modified polyglycerol-phosphate chains for phage adsorption.

A mutation frequency of 32% was observed in the *06875* gene during the phage CP39 infection, indicating that the pressure of a specific phage would allow a rapid phase variation due to the frame shifting, and developed the phage-resistant subpopulation in *C. jejuni*. In addition, mutations randomly occurred in different sites of the *06875* gene, including the point mutation or replacement of a short sequence, but no mutations were located in homopolymeric tracts as with previously reported genes encoding MeO*P*N transferase [12,19]. Besides the *06875* gene, several SNPs were also observed in genes, including *03815*, *06335*, *06385*, *06420*, *06565*, and *06875*. Both *06335* and *06385* encode flagellate receptor-transporter protein PseD. The down-regulated expression of PseD could prevent the infection of a flagellotropic phage FLaGrab [33]. However, we did not observe any evidence that the infection of phage CP39 required flagella, as both Δ*motA* and Δ*flaAB* were sensitive to phage CP39. The rapid development of phage resistance and the complex defense mechanisms indicated that it is essential to understand the phage–host interaction before applying phages in food production.

## 5. Conclusions

Our study demonstrated that phage CP39 was a CPS-dependent phage belonging to the Myoviridae family. Phage adsorption required the *06875* gene encoding the CGPTase in *C. jejuni* NCTC12662. During the phage infection, a high mutation frequency of *06875* with random mutation sites would cause the rapid development of phage resistance by the phase variable expression of the non-functional protein. Therefore, we proposed that preventing or inhibiting phage CP39 mainly depends on the phase variable gene expression of *06875*, while the occurrence of SNPs in genes except for CPS loci also indicated a diversity mechanism of phage–host interaction. Our study provides additional information concerning the evolutionary adaption of *C. jejuni* against phages, which would be necessary before phage application in the industry.

## Data Availability

Publicly available datasets were analyzed in this study. The phage CP39 genome information can be found with accession number MH107028 in ENA database; The phage CP39 resistant isolates including 12662_CP39R1, 12662_CP39R2, 12662_CP39R3, 12662_CP39R4 can be found with accession number PRJNA798540 in NCBI database.

## Supplementary Materials

The following supporting information can be downloaded at: https://www.mdpi.com/article/10.3390/v14030485/s1, Figure S1: Growth curves of Δ*motA* and Δ*kpsM* mutant strain. The *motA* and *kpsM* mutant strain incubated in MH broth for 24 h. The same culture NCTC12662 served as the control; Figure S2: Sensitivity assay of mutant strains to CP39. (A) Sensitivity of 06875_492delT mutant strain to CP39. (B) Sensitivity of Δ*flaAB* to CP39; Table S1: Strains and plasmids used in this study; Table S2: Referece strains for the phylogenetic analysis; Table S3: Primers used in this study; Table S4: *C. jejuni* NCTC12662 genes with SNPs after phage CP39 infection.
