# Supplementary figures and images for: Campylobacter jejuni Developed the Resistance to Bacteriophage CP39 by Phase Variable Expression of 06875 Encoding the CGPTase
Source: Viruses. 2022 Feb 26;14(3):485. doi: 10.3390/v14030485 (PMC8949473; doi:10.3390/v14030485)

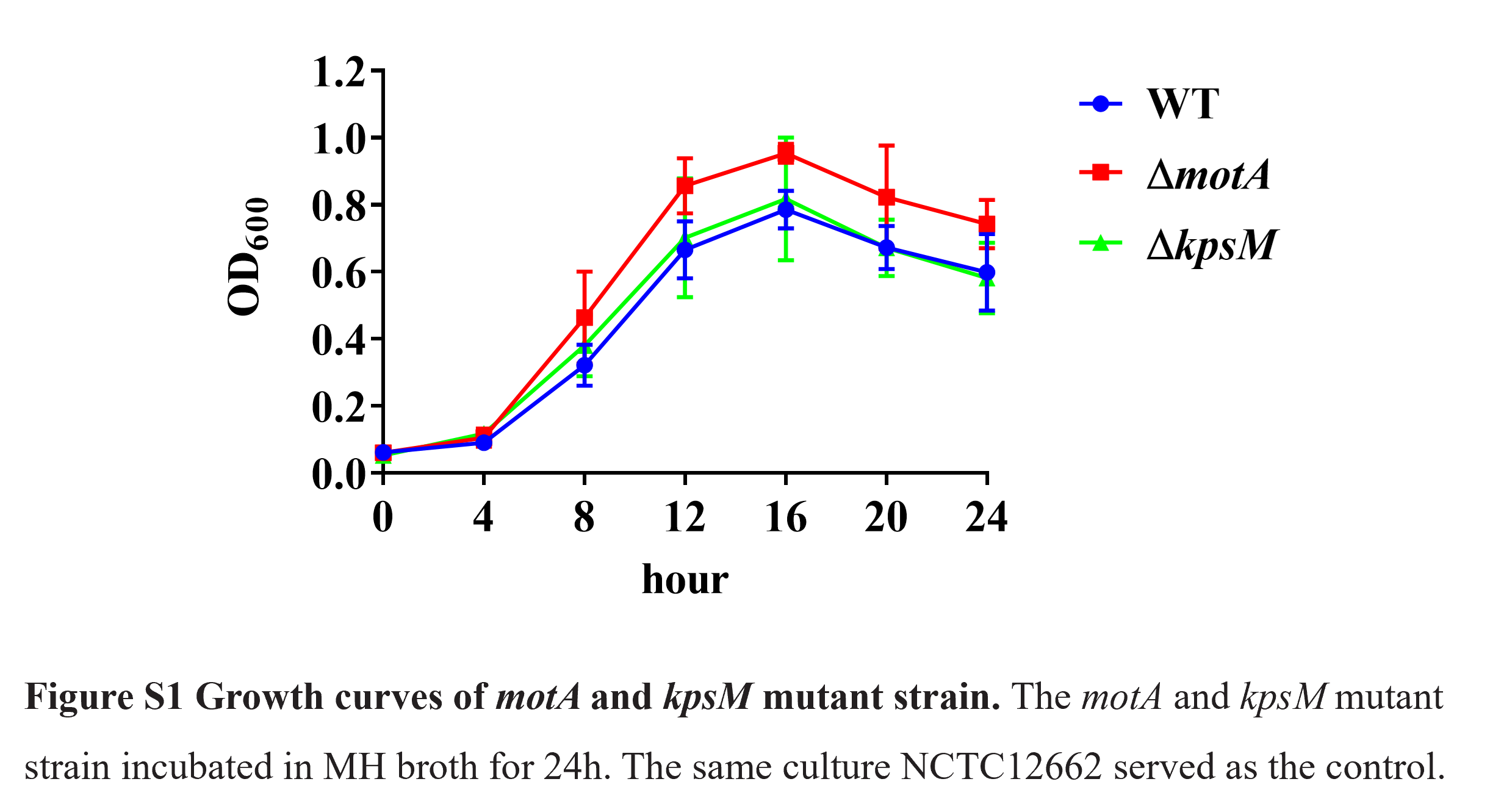

Supplement: Supplementary file 1 [file viruses-14-00485-s001.zip › supplementary-1592165/Supplemetary materials/Supplementary figures/Figure S1.tif]

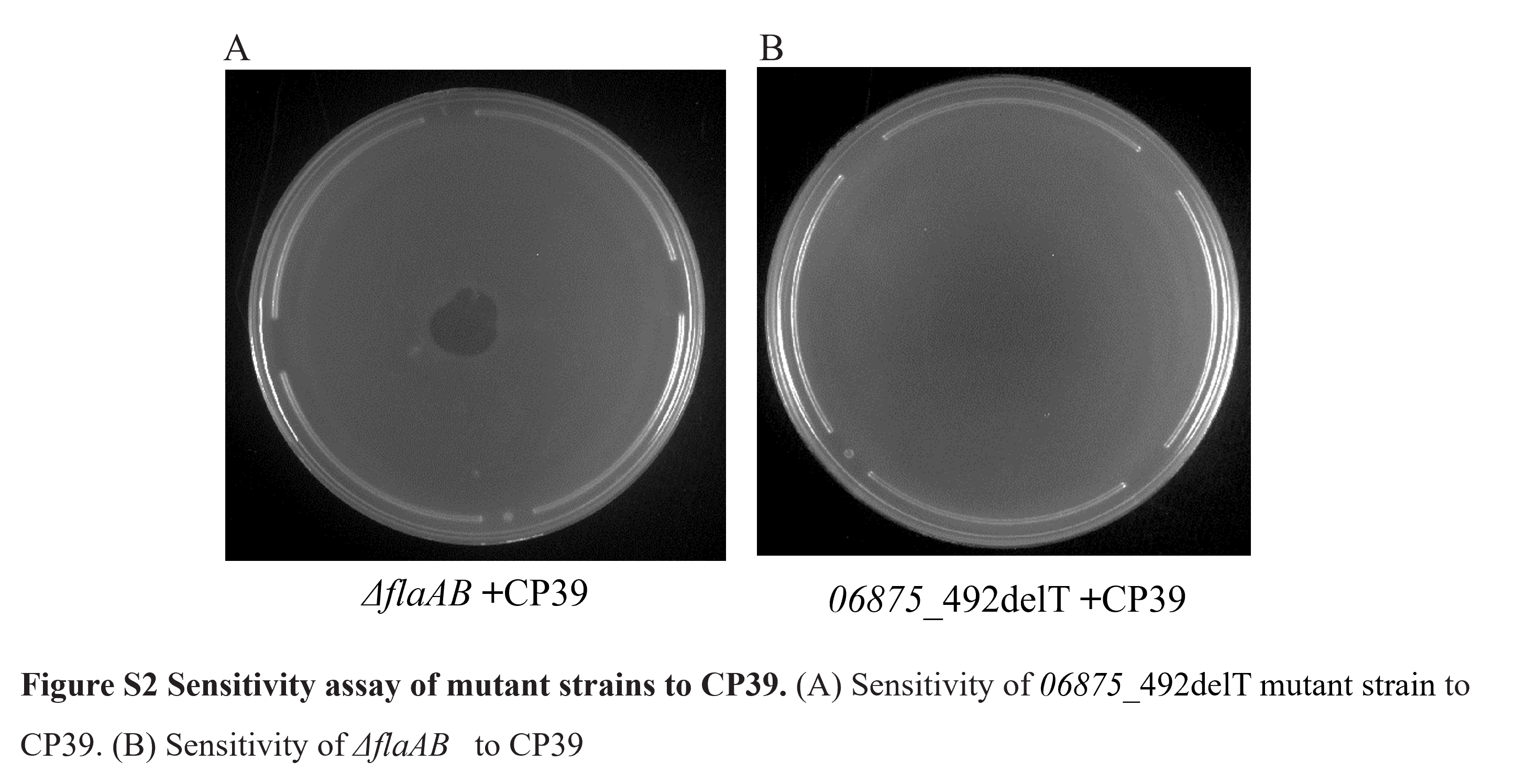

Supplement: Supplementary file 1 [file viruses-14-00485-s001.zip › supplementary-1592165/Supplemetary materials/Supplementary figures/Figure S2.tif]
